# Mechanical Dentistry

**Published:** 1842-09

**Authors:** 


*Mechanical Dentistry.*—The continuation of the article by the New York editor on mechanical dentistry is postponed until the next number, in consequence of the amount of other matter resulting from the recent meeting of the American Society of Dental Surgeons. We are gratified to learn, from various and highly respectable sources, that the publication of this treatise is well received. That it has already been productive of good, we have the most satisfactory assurances. Little, comparatively, has been written on this branch of dental surgery, and this will supply the readers of the Journal with information that can be procured from no other publication. That the manipulations pertaining to this department of the art can, in this way, be thoroughly taught, we would not affirm; but there are many who are skilled in the manipulations, but are ignorant of the plan or principle upon which artificial teeth should be adapted and secured in the mouth, and to such the present publication will be eminently useful. H.

